# Is self-advocacy universally achievable for patients? The experiences of Australian women with cardiac disease in pregnancy and postpartum

**DOI:** 10.1080/17482631.2023.2182953

**Published:** 2023-02-23

**Authors:** Jane Hutchens, Jane Frawley, Elizabeth A. Sullivan

**Affiliations:** a School of Public Health, Faculty of Health, University of Technology Sydney, Ultimo, Australia; b Faculty of Health and Medicine, University of Newcastle, Newcastle, Australia

**Keywords:** Cardiac, self-advocacy, pregnancy, postpartum, voice, gender

## Abstract

**Purpose:**

Patient self-advocacy is valued and promoted; however, it may not be readily accessible to all. This analysis examines the experiences of women in Australia who had cardiac disease in pregnancy or the first year postpartum through the lenses of self-advocacy and gender, specifically seeking to elaborate on the contexts, impacts, barriers, and women’s responses to the barriers to self-advocacy.

**Method:**

A qualitative study design was used. Twenty-five women participated in semi-structured in-depth interviews. Data were analysed using thematic analysis.

**Results:**

Analysis of findings generated the following themes: 1) Silent dream scream, 2) Easier said than done, 3) Crazy-making, and 4) Concentric circles of advocacy. Regardless of women’s personal attributes, knowledge and experience, self-advocating for their health was complex and difficult and had negative cardiac and psychological outcomes.

**Conclusion:**

While the women encountered significant barriers to self-advocating, they were resilient and ultimately developed strategies to be heard and to advocate on their own behalf and that of other women. Findings can be used to identify ways to support women to self-advocate and to provide adequately resourced and culturally safe environments to enable healthcare professionals to provide person-centred care.

## Introduction

Is self-advocacy universally accessible, achievable or safe for patients? Health policymakers, patients, advocacy groups and health professionals advocate for a shift from medically dominant care models to related conceptual models of person-centred care, woman-centred care in maternity services, self-managed care, shared-decision making, and self-advocacy (McCorkle et al., 2011; Timmermans, 2020). A move away from paternalistic communication and disproportionate power relations requires the patient to be engaged (Timmermans, 2020), self-empowered (Wahlin, 2017), able to self-advocate (T. L. Hagan & Donovan, 2013; Thomas et al., 2021), to assume more responsibility, and to be more active in their care and recovery (Lundmark et al., 2016). These requirements highlight the complexity of shifting paradigms.

Patient self-advocacy has been defined as “*gaining and using knowledge to assertively communicate and make decisions*” (Brashers et al., 1999) and a patient’s behaviour and ability to get their needs met in the face of a challenge (Clark & Stovall, 1996; T. Hagan et al., 2017). These definitions infer that self-advocacy means getting one’s preferences met, regardless of circumstances; however, effective self-advocacy is grounded in communication more than outcome, and this distinction may be even more important when there is a lack of guidance on management options, and in an emergency such as a cardiac event. Accordingly, this paper adopts a process-rather than an outcome-focused definition of self-advocacy as “*representing one’s own interests within the health care decision-making process” (Wright et al., 2007, p. 36)*.

Research on gender and women’s experiences of self-advocating has been conducted in gynaecological and maternal care (Young et al., 2015), and in the management of pain (Kolmes & Boerstler, 2020). Disenfranchised populations, including women with uncommon medical conditions, are more vulnerable and may be less represented within the dominant culture or systems, leading to additional challenges attempting to self-advocate.

The benefits of effective self-advocacy include improved person-centred care and quality of life, and reduced symptom burden and use of preventative health services (Thomas et al., 2021). Research indicates a positive correlation between patient self-advocacy and patient satisfaction (Brashers et al., 2017; Senders et al., 2016). A secondary benefit may come from confirmation of patients’ ability to influence care, increasing their confidence to address other areas, potentially reducing inequities and disparities they may encounter within the health care system (Thomas et al., 2021). Lastly, in currently under-researched areas, healthcare professionals (HCPs) and other patients stand to benefit from an improved understanding of patient perspectives.

### Cardiac disease in pregnancy and the first year postpartum

This article examines the self-advocacy experiences of women with cardiac disease in pregnancy and the first year postpartum (CDPP). CDPP includes a heterogeneous group of acquired, congenital and genetic conditions, including structural heart and aortic disease, cardiomyopathies, rhythm disorders, ischaemic heart disease, and arterial dissections. Based on a prevalence of 1% to 4%, (Regitz-Zagrosek et al., 2018) there are 1.3 to 5.2 million women affected by CDPP globally, of which 3,150 to 12,600 are Australian women (Australian Institute of Health and Welfare, 2021). Current estimates of prevalence, morbidity and mortality are likely to under-ascertain the disease burden (England et al., 2020; Vijayaraghavan et al., 2014).

There is little data on the healthcare experiences of women with CDPP, including their experiences of self-advocating. Research on the experience of being diagnosed with CDPP describes women losing trust in the health system after having their symptoms dismissed by HCPs, causing significant distress (Dekker et al., 2016; Patel et al., 2016). Other studies examined mental health and CDPP (Liang et al., 2014; Pfeffer et al., 2020) and birth trauma and the experience of being acutely unwell and hospitalized as a mother of a newborn (Power et al., 2015).

### Self-advocacy and cardiac disease in pregnancy and postpartum

Women with CDPP have lifelong cardiac conditions which may reduce the quality and length of their lives (Koutrolou-Sotiropoulou et al., 2016; Rosman et al., 2019). CDPP is a time and context-defined chapter; however, cardiac health is not static and as circumstances change, women need to adjust and re-negotiate care and their daily lives. They interact with a range of HCPs over time, each with their own clinical focus, communication and management style, and women are required to establish functional productive relationships with them all, often at times of illness, acuity and distress. The complex management required to promote and protect their physical and mental health places significant demands on women (Asbring & Närvänen, 2004). Further, women with CDPP need to make informed decisions in an environment of little research, limited and at times conflicting guidance, and without the same level of organized support or access to peers as those with more common conditions. It is important to understand women’s experiences of self-advocating in this context to inform future education and support services.

The aim of this analysis is to examine the data through the lenses of self-advocacy and gender, specifically seeking to explore and elaborate on the contexts, impacts, barriers and women’s responses to the barriers to self-advocacy.

## Method

### Study design

We used in-depth semi-structured interviews to understand women’s experiences of CDPP. Qualitative research in the health field provides insight into patient experiences that not only validates their stories but also raises awareness, and makes meaningful change possible (Denzin & Lincoln, 2011). The concept of the study was discussed with clinical and community groups from the NSW Heart Foundation.

### Setting

Australian healthcare is underpinned by Medicare, a universal public health and medical insurance scheme funded by taxpayers. Public patients in public hospitals receive free treatment but are unable to choose their doctor. Public outpatient clinics are available; however, specialist services are restricted to major metropolitan hospitals and face resource limitations. Some services are only available in the public system, such as specialist genetic cardiac disease clinics. Private healthcare includes private hospitals and specialist services. Privately insured patients may have access to health fund rebates for attending private cardiologists or obstetricians. At the time of writing, there are two part-time public cardio-obstetrics clinics in Australia, and they do not cater for women diagnosed with cardiac conditions in the first year postpartum. Likewise, obstetric physicians are available in some large public hospitals but not all, and they care for women during pregnancy, not up to a year postpartum.

### Sample

The population we sought to interview was hard to reach as it involves uncommon conditions. There is also a lack of services specifically for CDPP, and limited disease-based support groups. Online recruitment has been demonstrated to be effective in accessing hard to reach groups and thus was our main approach (Whitaker et al., 2017; Wozney et al., 2019). Recruitment proceeded via posts on the social media accounts of selected consenting pregnancy and parenting groups and cardiac support groups, and invitations distributed by cardiac support groups to members’ emails and or group newsletters. The women were purposively recruited because they are, or have been, mothers with a diagnosis of cardiac disease who were willing to participate in an in-depth interview. Recruitment was from December 2018 to April 2020, when we had adequate data to sufficiently describe and analyse the women’s experiences, and answer our research question (Braun & Clarke, 2021b).

Thirty-three women responded to the recruitment posts, two did not meet the inclusion criteria, six were lost to follow-up, and 25 were interviewed. The women had congenital (*n* = 5), genetic (*n* = 9) and acquired (*n* = 12) cardiac disease in pregnancy or the first 12 months postpartum and gave birth in Australia to at least one live-born baby of 20 weeks gestation or 400 gm birthweight (noting that one woman had a genetic and a congenital condition). The women had pre-existing diagnoses (*n* = 9; diagnosed from 2 days old to 26 years old), antepartum diagnoses (*n* = 6) and postpartum diagnoses (*n* = 10; diagnosed from 2 weeks to 11 months postpartum). The majority were first-time mothers (*n* = 15), 5 had their cardiac diagnosis associated with the 2^nd^ pregnancy, 4 with their 3rd pregnancy and 1 with her fourth pregnancy. Most women lived in metropolitan areas, and of the four that lived in regional or rural areas, two transferred to metropolitan hospitals for care during their pregnancy or postpartum event. Fifteen women (60%) had tertiary level education, 7 (28%) had trade level and 3 (12%) had high school education. Their median age at interview was 39 years (range: 28–59). The time from their CDPP to the time of interview was median 36 months, mean 5 years 7 months. The women’s characteristics are outlined in Appendix A, and their diagnoses are listed in Appendix B.

### Data collection

Data were collected using individual semi-structured, in-depth interviews, a recognized method for investigating topics about which little is known. Semi-structured interviews can accommodate diverse perceptions and enable women to share the issues that are meaningful and important to them (Cridland et al., 2015). The interviews were conducted by phone and by a single interviewer (JH). Interviews began with confirmation of informed consent and the collection of basic demographic data. Personal details including names and addresses were not recorded with the study data. Information was re-identifiable only to the interviewer. The interviews took between 24 and 90 minutes and with the women’s permission interviews were audio-recorded or hand-transcribed verbatim, including notable non-verbal responses such as crying or laughing.

The lead question of the interview guide was “Can you tell me about your experience?” Further open-ended questions were devised during the interviews in response to the woman’s story and where indicated prompts were used to clarify and expand on the story. This style facilitated an open discussion and encouraged the women to direct the narrative of their story, and include feelings, attitudes and reflections in their own words.

### Analysis

Interpretive inductive data analysis was performed using the reflexive thematic analysis (RTA) approach by Braun and Clarke (Braun & Clarke, 2006, 2021a). RTA was used as it is theoretically flexible and accessible, positions the researchers’ subjectivity as a resource, and allows for thick description, interpretative story-telling and nuanced theme development (Braun & Clarke, 2006, 2019; Gillberg & Jones, 2019).

Informed by the six stages of analysis outlined by Braun and Clarke (Braun & Clarke, 2006, 2021a) (familiarization, code generation, theme development, reviewing and refining themes, defining themes and report writing), data coding and theme development was organic and responsive to developing patterns. All study team members listened to the interviews and read the transcripts. JH led the analysis by immersing herself in the data and developing and refining codes and themes by selecting illustrative quotations.

Quality was determined using the guidelines provided by Braun and Clarke (Braun & Clarke, 2021a). In particular, the researchers engaged in ongoing discussion, reflection and development of the codes and themes, exploring individual and shared perspectives on the patterns within and across the women’s stories and reflecting on our influence on interpreting and reporting the data.

The researchers are female healthcare professionals with diverse sexual and reproductive health and public health experience. The approach to analysis was broadly informed by feminism, critical theory and social constructionism (understanding the multiplicity of realities which are constructed through our interactions with others, and situated within social structures that determine power and oppression) with latent themes (reflecting the underlying assumptions and meanings of the stories and semantic meanings) (Braun & Clarke, 2006; Creswell & Poth, 2018).

### Ethical considerations

This study was approved by the University of Technology Sydney’s Human Research Ethics Committee (ETH19–3372), in accordance with the *Australian Code for the Responsible Conduct of Research* and the *National Statement on Ethical Conduct in Human Research*. The participants received oral and written information about the aim and procedure thereof and were informed their participation was voluntary and that they could withdraw at any time without specifying any reason. Consent was obtained from all participants and all data were handled in a confidential manner.

## Results

Analysis produced four themes: 1) Silent dream scream, 2) Easier said than done, 3) Crazy-making, and 4) Concentric circles of advocacy. There was not a one-directional linear progression through the themes; instead, their experiences relating to self-advocacy were iterative and varied with circumstances and contexts. Some interactions were during labour and birth, others during the process of receiving a cardiac diagnosis and treatment, and others were during ongoing health care in the community.

### Theme 1: silent dream scream

All the women experienced the loss of voice, or of being silenced, by themselves, their loved ones and or by HCPs. They interacted with a diverse range of HCPs from a range of specialities and sub-specialities and the issues identified are distributed across these.

Women were silent and felt silenced, especially in the early days following diagnosis and during labour and childbirth. Feeling shocked, confused, fearful or in initial denial of their situation, some women were silent and hid their thoughts and feelings from their HCPs.

I was shaking in his room. I went down into the car and cried my eyes out … I was shocked. I didn’t let on though.

(HCM, 50 yrs)

Not wanting to identify with or be stigmatized as someone with a cardiac condition influenced some women to continue varying degrees of self-imposed silence, in one case “for 8–10 years I even avoided even communicating it.” (PVO, 48 yrs)

Some women felt silenced by their families who minimized the significance of their cardiac event and diagnosis, and who rejected advice or concern about potential risks as genetic relatives of the women.

We cancelled the ambulance because my husband was saying “you’re okay, you’re talking, you’re okay”, as men do. I was like, sure, because I didn’t want it to be anything serious either.

(PSCAD, 37 yrs)

The women felt they were silenced, ignored, and dismissed by HCPs, some over many years. These instances ranged from a “don’t you worry about that” type of response which was perceived as glib in the absence of meaningful discussion, through to feeling their spoken words were silenced and lost in a vacuum of impotence. A woman with lifelong positive cardiac healthcare experiences and who had previously felt that she used her voice and self-advocated successfully, found the situation different during labour and birth. She reported having been advised by her cardiologist and an anaesthetist that they were happy for her to attempt a natural birth without intervention, however, when she presented to the hospital in early labour her cardiologist was not there and was not consulted.

So, they rushed an anaesthetist in and gave me an epidural. Basically ordered me to, and I had the epidural and, I mean, you know, you’re scared, so you just do what you’re told, basically … they did the epidural, I was literally crying, saying that I don’t consent to this. You know, and they did it.

(CHD, 32 yrs)

Another woman who had worsening dependent oedema, shortness of breath and discomfort, and who was subsequently diagnosed with PPCM, sought an induction initially at 40 weeks before finally being induced at nearly 42 weeks. She reported asking to be induced several times due to her worsening undiagnosed cardiac symptoms, and two failed cervix sweeps, to be told that the hospital practice was that she “had to be 10 days over” to be induced.

I was begging them, I was like, can you please just induce me, and they refused.

(PPCM, 28 yrs)

### Theme 2: easier said than done

The women in our study reported similar challenges self-advocating, regardless of cardiac condition or category, or if their experiences were during pregnancy or postpartum. However, not all women experienced difficulties in all contexts and times. For example, some of the women with pre-existing cardiac disease had previously self-advocated effectively and felt part of decision-making processes; however, when they were pregnant and labouring, that autonomy and control was felt to be absent. This introduced another dimension to their loss of voice; that this new experience was so different to previous experiences and their expectations of the current healthcare encounter. Women had realistic expectations but were still disturbed by the extent of their loss of voice, and control.

I didn’t expect to have a sense of control because … it’s childbirth … if you try and control it, you know, you’re going to end up disappointed, but I *had absolutely no* control over any of it.

(HCM, 50 yrs)

For the women interviewed, this perceived lack of autonomy and control was in part due to the medical situation and in part because the women’s wishes or input were not incorporated into care planning, and when she sought information, she was at times met with silence. Women with cardiac disease that was pre-existing or diagnosed during pregnancy typically had multiple medical specialists and care was often perceived as uncoordinated. Women attempted to negotiate and liaise, but without any power or authority.

It was hard to make decisions mid-labour and hard having to self-advocate about doing what the cardiologist had instructed.

(HCM, 50 yrs)

The majority of women had little experience of advocating for themselves in a health context. It usually took enhanced distress to provoke their assertiveness. The following woman had a PSCAD during pregnancy and was separated from her baby immediately after giving birth.

But by the next morning … my whole personality changed. I was an absolute raving lunatic … I was very meek and mild as a patient [but was now] … a tigress trying to find her child.

(PSCAD, 44 yrs)

Some women blamed themselves when their health care experience was negative; for not advocating in a system that is not designed for such behaviour, and against socialization that, despite some changes, is still grounded in deference not challenge or shared decision-making. One woman’s husband suggested pushing for a transfer to a larger hospital.

I was saying “No, we should just let the system work. You know, the system should work.” And now I think “No” I should have said yes.

(PSCAD, 37 yrs)

Perceiving that you do not have rights in healthcare and that you are passive recipients of care prevented women from self-advocating.

Had I known about the Patient’s Charter, I might have known that it was ok to talk to her senior and say, like “is this right, that I should be staying up all night turning this alarm off because something is not right here.” I didn’t know that I could say that.

(HCM, 50 yrs)

### Theme 3: crazy-making

Feeling silenced, and that they had no sense of control, had significant impacts for women, far beyond their cardiac outcomes. When women perceived their concerns were dismissed or invalidated by HCPs, they were fearful of the possible health outcomes but they also started to doubt themselves and question their understanding of their own lived experiences.

I was starting to doubt whether I had the pains. And I thought, no, I actually did. I said to [my partner], “Did I actually have the pains?” And he said, “Yes, you had the pains” because I’m thinking oh my God, you know, this dismissiveness.

(PSCAD, 39 yrs)

This woman was transferred between hospitals to have an angiogram and “when they did the handover it was basically “this is just stress”’. The angiogram showed that she had had a PSCAD and heart attack, and while she was relieved to have a diagnosis;

I just went oh, you bastards … [the angiogram] almost validated that pain that I had. Because I thought “shit, is this in my head?” Am I making this up?

(PSCAD, 39 yrs)

Feeling that they were being dismissed and having their lived experiences denied had a pernicious effect, “It really, really made me feel like I was going insane.” (CHD, 32 yrs) Some women described being “brushed off” for years before their situation was acknowledged. This caused women to fear death and uncertainty, having a marked effect on their mental health, self-confidence and self-belief.

Oh, it was terrible. I think it was worse than everything put together. I felt like they were telling me that I was going crazy. I was so lucid.

(PSCAD, 44 yrs)

Women’s experiences of feeling silenced and out of control in healthcare interactions were compounded when they sought medical and mental health care to manage these feelings. One woman who had post-traumatic stress disorder (PTSD) and depression following her birth trauma described her experience:

I kind of felt like everyone I spoke to except for my GP justified what had happened at the hospital. And that kind of made me feel shit, because it made me feel like I was going crazy … It was very invalidating, because I mean, it was my lived experience.

(CHD, 32 yrs)

### Theme 4: concentric circles of advocacy

In time, women’s experiences of advocating involved advocating for themselves, their children, and for some, other women and girls with cardiac disease.

### My self

Some women were able to advocate from the outset on issues that they had knowledge of. One woman who had, at that point, little knowledge about her condition, was less able to advocate for her health compared to her clarity about ensuring her newborn went in the ambulance with her when she was transferred to a different hospital: “I said ‘I’m breastfeeding, she comes with me’, and they were like ‘okay’”. Then when she arrived at the second hospital,

… they kept saying we may have to send your baby up to paediatrics, won’t be able to stay with you. I said, “well, that’s not happening”.

(PSCAD, 38 yrs)

Women who had subsequent pregnancies used their experience and knowledge to self-advocate, negotiate and assume some control: “The second time I was able to say ‘I don’t want one of those’” (HCM, 29 yrs) and “This time I told them not to give me drugs of any sort until I came out” (LQTS, 30 yrs). One woman self-advocated during her second labour regarding the recommended medication loading dose for her induction in a way she was unable to with her first birth.

“Okay, but I don’t want it”. So my [independent] midwife kind of bargained with them and said, “Why don’t we start it on a half dose? If nothing happens, we can up the dose”. I was like “yes, I’m happy with that.”

(CHD, 32 yrs)

This is also an example of women building their advocacy team, including recruiting other HCPs and partners to the role of temporary vessels of her voice when she may be unable to access her own agency; “I trained him to within an inch of his life; he was … able to advocate if needed.”

Women developed their agency and advocacy by seeking second opinions, increasing their knowledge, and by being clear about their expectations and what was and was not acceptable. Women also learnt about their rights, including being able to ask for interventions to stop:

In Emergency, a doctor put a central line in via the groin but I had to tell him to stop because it was obvious he didn’t know what he was doing. They’re learning but as a patient, you’re also learning your rights.

(HCM, 50 yrs)

One woman’s strategy to ensure her voice was heard was to keep a logbook of her pulse rate, blood pressure, oxygen saturation, weeping oedema and symptoms such as shortness of breath and orthopnoea. She was knowledgeable about her condition and had previously had her shortness of breath and central cyanosis dismissed by an emergency department resident as due to a heavy menstrual period and not the significant cardiac compromise she was experiencing. Following this and other incidents where she felt she was not heard or assessed accurately, she “kind of had to take charge of [her] own health” and aimed to bolster the authority of her voice by keeping a logbook:

When I went back to the congenital cardiologist, I had documental proof, saying, “This is what’s been going on.” And then they listened.

(CHD, 30 yrs)

Women’s cardiac conditions were uncommon and often rare during pregnancy and the first year postpartum. The women needed to self-advocate to assume some control of their health, in a way they may not have if their condition was well understood.

You have to be very knowledgeable about your own condition, because people don’t know about it. They’ve never heard of it … You’ve got to be proactive about that. Most of the cardiologists at the hospital probably wouldn’t know anything about this condition.

(LQTS, 38 yrs)

Women learnt about their cardiac condition in online support groups, which helped them discuss their concerns with their cardiologists.

… since I’ve found this support group, I knew what questions to ask. Before then, I’d been riding the road of the blind. I had no idea what questions to ask. But slowly and surely, after listening to all these other SCAD sufferers, I’ve thought, “Oh, I wonder what a LAD is and where my tear was, and I wonder how much damage.

(PSCAD, 44 yrs)

Being able to self-advocate and assume some sense of autonomy and control also helped women’s mental health and quality of life.

I’m a bit more proactive, I go in with a list of questions … “Is there anything that you need to know about how I’m feeling in relation to my ongoing health”, and they’ll say “No. Not really”. And, I’ll say “Well, have there been any advances in medical research recently?” The answer is no always. But I continue to ask the questions, because it helps me to keep going, to know that maybe one day they will actually invent a cure for this.

(LQTS, 38 yrs)

### My child

Mothers advocated for their children to be tested, to be safely breastfed, and if the children had a cardiac condition, for them to be treated according to guidelines. They “had to run a bit of a squeaky wheel campaign” (LQTS, 38 yrs) to ensure their baby was tested for genetic conditions.

They were insistent when it came to their child’s safety; “Something’s not right. You need to go and get someone” (CHD, 32 yrs), and challenging cardiac care for their child; “No. No. That’s not what the recommendations are for this disease.” (HCM, 44 yrs) They rejected recommendations to bottle feed for ease and worked with the doctor and pharmacist until they “ … found something that was safe for breastfeeding because I was quite insistent on that”. (PSCAD, 38 yrs)

### My community

As women recovered, some added the broader community to their advocacy efforts. A woman who repeatedly implored the ambulance staff to take her to a tertiary referral hospital was instead taken to a small general hospital where she stayed for 48 hours, not under the care of a cardiologist. In this time her PSCAD and MI were undiagnosed and unmanaged, incurring further cardiac damage. Once recovered enough to engage, she provided feedback to the ambulance service. Instead of seeking a formal apology or suggesting performance management for the staff involved she negotiated that they provide further training so that they were more aware of the characteristics of cardiac disease in young women, especially in pregnancy and postpartum, including PSCAD.

Women found their voices and transitioned from being a member of a Facebook support group to moderating and managing the group. Some women also posted their stories on their private social media pages to increase community awareness of cardiac disease in pregnant and postpartum women, and younger women in general which generated discussion and prompted readers to attend to their health differently.

Women found their voice through a range of community and health projects; they undertook fundraising for equipment and research, ran and contributed to awareness campaigns, and were interviewed in local and national media to increase awareness and to advocate for women. They were members of cardiac care reference groups, acted as peer support for other younger women with cardiac disease and ran peer support education. Being able to use their voice increased their confidence and sense of empowerment.

The women responded to the invitation to be interviewed for this study to have their stories heard; they did this for themselves, but a stronger motivation was to advocate for more research and better care for future girls and women.

Yes, it was pretty traumatising but it just made me more determined in my work, to not allow that for anyone else.

(CHD, 32 yrs)

When asked what motivated her to participate a woman in her mid-20s replied that despite having cardiac conditions;

… it’s really important for women to still be empowered and have a say in their pregnancy and their labour.

(CHD, 28 yrs)

Even if their physical and emotional healing was not complete, they had reached a place where they were using their voice.

## Discussion

This article examined the concepts of voice and self-advocacy in the lived experiences of women who had cardiac disease in pregnancy and the first year postpartum. Women faced multiple and significant barriers to self-advocacy despite being articulate and educated. They reported times when they were hesitant to self-advocate; however, the more pervasive experience was having their efforts to self-advocate dismissed. The difficulties in having their voices heard and responded to constructively had a marked negative effect on their emotional well-being. The barriers the women faced can be understood at an individual, interpersonal, and societal level.

The following discussion considers self-advocacy for this population in terms of personal attributes for self-advocacy and broader societal gender perspectives.

### Personal attributes for self-advocacy

Both the experience and effectiveness of self-advocacy are mediated by numerous variables including individual traits, past experiences, availability of information, and the HCPs and health system women interact with. The literature on self-advocacy identifies numerous prerequisites and barriers to self-advocacy. In brief, individuals require; 1) Connection: access to, and disposition to utilize support systems; 2) Communication: the ability to communicate effectively with a diverse range of health professionals; and 3) Knowledge: the capacity to access, evaluate and use health information (T. Hagan et al., 2017; Waddell et al., 2021). Assertiveness and “mindful nonadherence” (Brashers et al., 1999) as well as being able to practice self-compassion to buffer any negative consequences of being assertive or seeking alternatives (Ramos Salazar, 2018) are additional criteria. This brief synopsis of personal attributes belies the complexity of self-advocating at all, let alone in an acute health situation such as childbirth or during a cardiac event or a composite of both scenarios.

The women in our study were uniquely vulnerable as they had significant cardiac disease and acute cardiac events while they were pregnant or a new mother. Both the cardiac and the maternal circumstances are challenging and associated with trauma and distress and having both amplified that effect. A few women with pre-existing conditions were able to access specialist care with an obstetric physician or foetal-maternal medicine physician, but for most, care was fragmented as they tried to coordinate their care between subspecialties. Women with new diagnoses postpartum felt untethered, trying to coordinate care between their cardiologist and general practitioner. No-one felt they belonged regardless of where they were. Most women were not referred to rehabilitation or support groups or informed of Facebook support groups, and it took time for them to find connections, especially for rarer conditions. No cardiac support groups were specifically for mothers, consequently their support and connection needs were only partially met. Most of the women described feeling completely wrong-footed and even disoriented in the early stages, feeling at times, profoundly isolated. The shock, distress and trauma, sparse information and the lack of a community of peers from which to draw strength are counter to the required attributes described by T. Hagan et al. (2017) and contribute to the reasons these capable, competent women still had difficulty self-advocating.

Self-advocacy, person-centred care and shared decision-making are interlinked, requiring strong bi-directional communication. It is relevant to reflect on the background of the women in this study; most were tertiary educated, many were in senior roles, four were health care professionals and one was a health manager. All women in this study spoke fluent English and engaged in lengthy in-depth interviews, clearly articulating their stories. Yet they all experienced difficulties in self-advocating when they had cardiac disease in pregnancy and postpartum, typically on multiple occasions. Given this cohort of patients had challenges with self-advocacy, and by extension with person-centred care and shared decision-making, it is probable that others would face greater barriers, including individuals with low literacy and language skills (Légaré & Witteman, 2013; McCormack et al., 2017; T. L. Hagan & Donovan, 2013).

The efficacy of self-advocating was lowest, and self-silencing and the sense of being silenced were most pronounced during the high acuity situations of labour and acute cardiac events. Our findings were consistent with previous research reporting that in the early stages following severe maternal morbidity some women “ … wanted to bide their time and heal and some wanted a voice in their healthcare” (Cram et al., 2019, p. 63). Thomas et al. (2021) described a constellation of circumstances for women with cancer to be able to self-advocate, including developing personal skills and adjusting their priorities. For women in our study the critical time to self-advocate was in high-acuity situations where there was little or no time to gather information, consult widely, reflect or seek connection.

Once the acute stage had passed, women still faced fundamental challenges in self-advocating. Their conditions were uncommon or rare, and data to inform shared decision-making and efforts to self-advocate was scarce. The majority of women were not attempting to self-advocate for different treatment options or interventions in this knowledge void; rather, their advocacy efforts and goals were often rudimentary. They wanted to be heard. And being heard would facilitate being assessed, diagnosed and treated with the degree of attention and urgency their conditions required. Women wanted to be told what their diagnosis was and to have it explained to them; to be provided with what evidence there was and when there was none, to have the rationale of recommended care explained.

Effective communication is predicated on bi-directional knowledge transfer, to both gain knowledge and build relationships. Silencing, as described by the women in our study as they attempted to describe their symptoms, values and needs represents missed opportunities to provide person-centred care and to add to the knowledge bank and understanding of rare and uncommon conditions. Knowledge is not neutral; what is known, by whom and what value that knowledge has is fundamentally embedded within power relationships, equity and equality (Gillberg & Jones, 2019). Patients may inadvertently contribute to the one-sidedness in knowledge exchange by undervaluing the significance of their expertise in their own body and experience, and underestimating their ability to share knowledge that HCPs “own” (Joseph-Williams et al., 2014). Lastly, while power is constructed as knowledge (Foucault, 2007), it is such only if wielded; if women were not able to self-advocate even in the times when they had objective knowledge, then they remained without power.

### Gender and self-advocacy

The imbalance in ownership and valuing of knowledge exists in a socio-political context that diminishes women’s knowledge and lived experiences, including of illness (Cole, 2021; Werner & Malterud, 2003). Women’s experiences in other settings influence their interactions and ability to self-advocate in the health setting, in particular any previous experiences or expectations of backlash against assertiveness or self-advocacy. Such backlash against women is theorized as retribution for violating gender norms, acting counter to beliefs about proper roles for males and females and disturbing the rightful social order (Rudman et al., 2012; Williams & Tiedens, 2016).

Occupational research indicates that women who self-advocate are viewed as less likeable and not as warm and that women hedge their assertiveness to minimize backlash and maintain a positive perception of themselves. Similarly, some women in our study were motivated to explain that they were normally a “the likeable patient, the good patient” (Amanatullah & Morris, 2010; Williams & Tiedens, 2016) and “not the kind of woman who complains” (Werner et al., 2004). Women self-advocating with male specialists are subverting social order on multiple dimensions; as women, as patients, as mothers, and possibly as people of perceived lower status. Women may be more able to self-advocate with female specialists who may be more likely to practice patient-centred care.

Silencing and infantilising women are not new. In *The Public Voice of Women: Oh do shut-up dear* Beard (2014) traces the first Western recorded ownership of speech, knowledge and power in Homer’s Odyssey when Telemachus tells his mother, Penelope, to go to her room because “*Speech will be the business of men, all men and me most of all, for mine is the power in this household*”. Gendered silencing can be overt and even violent; however, it can also be subtle and under the guise of paternalistic care. This “benevolent sexism” (Travis et al., 2012) can be seen in our study for example when women asked simple questions about what their diagnosis and treatment plan was and described being dismissed with comments like “don’t you worry about any of that”.

### Gender and cardiac disease

Women are under-represented in cardiac research (Jin et al., 2020; Norris et al., 2020) and when they are included, data may not be sex-disaggregated (Doull et al., 2010; Lam et al., 2019). Research on cardiac disease and pregnancy and postpartum is also limited. Women with cardiac conditions are under-diagnosed, under-treated and less likely to have interventions and treatment adherent to clinical guidelines (Arora et al., 2015; Bachelet et al., 2021; Johnson et al., 2018). Women are less likely to be referred to cardiac rehabilitation even though attendance may be associated with a greater reduction in mortality compared with men, noting there is limited research on pregnancy-related cardiac disease (Colbert et al., 2015; Colella et al., 2015; Sawan et al., 2022). Consequently, women with cardiac disease have worse morbidity and mortality outcomes (Alabas et al., 2017; Butters et al., 2021).

Research demonstrates that women’s cardiac outcomes are poorer if there is a physician-patient gender discordance (Lau et al., 2021). In contrast, Sun et al. (2021) found no gender discordance difference in length of stay or mortality but that female patients with a female surgeon (or a female surgeon and anaesthetist dyad) had shorter lengths of stay, confirming findings by Greenwood et al. (2018) that male physicians treating female patients was associated with poorer outcomes, not gender discordance per se. Education is recommended for healthcare professionals and for women to be empowered to self-advocate, however it is critical to ensure women do not experience negative fall-out if their efforts in self-advocating develop faster than education and cultural changes within which they seek care (Stehli et al., 2021). Further, recent research highlights the functions and benefits of a cardio-obstetric, multi-disciplinary team approach to cardiac disease in pregnancy, which may reduce some of the issues faced by the women in our study (Easter et al., 2020; Magun et al., 2020; O’kelly et al., 2021).

### Gender and reproductive health

Similar to women with cardiac conditions, women with reproductive issues experience being dismissed, under-diagnosed and misdiagnosed (Osborn et al., 2020). Gynaecological symptoms that are not readily diagnosable may be attributed to psychiatric disorders (Jones, 2015) and women’s knowledge of their own bodies again being taken as subordinate to the authority of doctors (Lupton, 2012; Kate Young et al., 2020). Women in our study consistently had their expertise in their bodies undervalued, whether it was about existing conditions or in describing symptoms of de novo conditions. In place of accepting the primacy of their bodily knowledge they were asked if they were an “anxious type”. The tendency to defer to sociohistorical constructions of women and their bodies (including hysteria discourse) may be amplified in situations of medical uncertainty, and this may be relevant in the case of young women with cardiac disease in pregnancy and postpartum (Lian & Robson, 2017; Kate Young et al., 2019).

The potential role of gender in the experiences of self-advocating in our study is significant but it is also noteworthy that perceptions of negative interactions were not universal and that some women described supportive care and being listened to. While awareness of gender bias and discrepancies in health care and research is growing, to date research has focused on the object of bias, women, and little has addressed the subject, the health care professionals (Alcalde Rubio et al., 2020). Specifically, a recent scoping review identified that the majority of research on gender bias was related to cardiovascular disease and that the focus was on strategies to increase adherence to existing guidelines to standardize healthcare (Alcalde Rubio et al., 2020). The women in our study employed multiple approaches in attempting to self-advocate which is in line with findings by Kolmes and Boerstler (2020). Similarly, Maslen and Lupton (2018) found that women were resourceful, active, and creative in accessing and using online information to enhance their experiences in clinical consultations. Notwithstanding the recognition of the impact of societal gender-based barriers, there is a need to undertake further research and develop strategies to facilitate self-advocacy.

In all contexts, trust influences communication and self-advocacy and is essential for safe patient care (Chandra et al., 2018; Frey, 2011). Most research on trust has focused on patients trusting the physician, which is clearly important, but also reflects the paternalism discussed and the markedly unequal valuing of the HCP’s knowledge and experience over that of the patient (Grob et al., 2019; Thom et al., 2011). That patient’s experiential knowledge is viewed as lacking credibility is both flawed and restricts the ability to gather clinical knowledge and establish trust and rapport (Frey, 2011; Nizzi, 2021). Patients will never be able to effectively self-advocate if HCPs don’t trust them, regardless of the patient’s self-advocacy credentials.

Any focus on and development of patients’ ability to self-advocate needs to be mirrored by action by HCPs and health care systems, and needs to recognize the broader socio-political context. Effective self-advocacy is associated with positive outcomes; however, the burden of communication and person-centred care cannot rest with the party with the least power, the patients (Thomas et al., 2021). Further, only focusing on self-advocacy and self-empowerment of the individual does so at the cost of ignoring systemic barriers (Coddington, 2017). Encouraging patients to self-advocate with HCPs and healthcare systems that do not meet their self-advocacy constructively and positively risks the diminution of trust, weakening of relationships, emotional fallout, passive or active disengagement and resistance to pursue recommended treatment (Schinkel et al., 2019). Whether it is in an acute or chronic health situation, it is exhausting to constantly convince others of your credibility (Werner & Malterud, 2003).

## Limitations and strengths

Methodological limitations include that the majority of interviewees responded to social media recruitment strategies and thus women not using social media are unlikely to be included in this study. The study may be subject to recall bias, both positive and negative, as time has passed since the experiences discussed. Further, it is not representative of a diversity of women and thus the critical issue of intersectional amplification of barriers has not been examined. We argue that the vulnerability and barriers experienced by women with CDPP are significant and complex, leading to psychological distress and delayed diagnosis and treatment. This is in the context of the women in our study being white, educated and able-bodied. How does one self-advocate as a person who has experienced, potentially lifelong, personal and systemic racism, who is differently abled, has a different social status, religion, or sexuality? The findings of research must be built upon in different contexts to better address the additional complexity of intersectional barriers and influences (Crensha 1991).

We also recognize that there is no “one” voice and this paper does not and cannot speak to every woman’s experience. Our sample included women with a range of diagnoses and this heterogeneity means there are small numbers in each category. However, to our knowledge, this is the first study exploring women’s experiences of self-advocating across a spectrum of CDPP, and this allowed for the inclusion of women with rare diseases who otherwise may not be included in research due to the low prevalence of these conditions.

## Conclusion

The experience of having cardiac disease in pregnancy and the first year postpartum can be distressing, disorienting and isolating. Regardless of women’s personal attributes, knowledge and experience, self-advocating for their health is complex and difficult. The inability of women to have their voices heard had negative cardiac and psychological outcomes. Women were resilient and resourceful in finding their voices and through digital media to find and build a community around cardiac disease in pregnancy. However, person-centred care places a requirement on health systems to provide an adequately resourced and culturally safe environment where healthcare professionals are supported to provide person-centred care.

## Data Availability

Research data are not shared. The data are not available due to privacy or ethical restrictions.

## Appendix A: Table I. Characteristics of participants

**Table:**

Characteristics	n (%)	Mean (range)	Median (months)
*Category of cardiac disease (primary diagnosis)*			
Congenital	5 (20)		
Genetic	8 (32)		
Acquired	12 (48)		
*Timing of diagnosis*			
Before pregnancy	9 (36)		
In pregnancy	6 (24)		
Postpartum (2 days to 11 months)	10 (40)		
*Time since CDPP at interview*		*5 years, 7 months*	
*Median time since interview*			*36*
*Age at diagnosis*			
0–1 year	2 (8)		
1–12 years	2 (8)		
13–19 years	1 (4)		
20–24 years	1 (4)		
25–29 years	3 (12)		
30–34 years	4 (16)		
35–39 years	9 (36)		
40 years and older	3 (12)		
*Age at time of interview*		39 (28–59)	
*Education*			
School certificate (year 10)	3 (12%)		
Trade qualification	7 (28%)		
Tertiary qualification	15 (60%)		
*Occupation*			
Hospitality	1		
IT	1		
Creative arts	2		
Clerical and administration	2		
Community/personal support workers	2		
Manager	4		
Professional	13		

## Appendix B: Table II. Participant diagnoses

**Table:**

**Congenital heart disease**
Bicuspid Aortic Valve (BAV)
Left Ventricular Non-Compaction Syndrome (LVNCS)
Mitral Valve Prolapse (MVP)
Patent Ductus Arteriosus (PDA)
Patent Foramen Ovale (PFO)
Tetralogy of Fallot (TOF)
**Genetic**
Arrhythmogenic Right Ventricular Dysplasia (ARVD)
Hypertrophic Cardiomyopathy (HCM)
Long QT Syndrome (LQTS)
**Acquired**
Idiopathic Cardiomyopathy (ICM)
Peripartum Cardiomyopathy (PPCM)
Pregnancy Associated Spontaneous Coronary Artery Dissection (PSCAD)
